# The dry lab microscopist or prompt microscopist: do we need them?

**DOI:** 10.1007/s12551-024-01250-1

**Published:** 2024-10-30

**Authors:** Filip Braet, Weidong Cai

**Affiliations:** 1 School of Medical Sciences (Molecular & Cellular Biomedicine), Faculty of Medicine and Health, The University of Sydney, Sydney, NSW 2006 Australia; 2 Australian Centre for Microscopy and Microanalysis, Faculty of Medicine and Health, The University of Sydney, Sydney, NSW 2006 Australia; 3 School of Computer Science (Biomedical & Multimedia Information Technology), Faculty of Engineering, The University of Sydney, Sydney, NSW 2006 Australia

**Keywords:** Artificial intelligence, Automation, Bioinformatics, Biophysical microscopy, Computational biology, Deep learning, Structural biology

## Abstract

In modern biological microscopy, the explosion of data volume and complexity highlights the urgent need for specialised data management support roles. While traditional microscopy focuses on visual data presentation, the rapid increase in big data acquisition and data mining demands advanced handling and analysis. This gap underscores the need for “dry lab microscopists” or data experts skilled in microscopy data management, software interoperability, and AI-driven solutions. Job markets reflect this demand, pointing to the necessity for dedicated training programs. Integrating these specialists into research institutions is crucial for addressing digital data challenges and maintaining high standards in data integrity and analysis. Their role is essential for advancing research in the data-driven era.

## Unlocking data information with the dry lab microscopist

To answer the question posed in the title, the answer is “a resounding yes”, as extracting quantitative information from big data is worth more than a thousand pictures. Since the early days of microscopic imaging good practices in data management, data analysis and data presentation are the core business of a microscopist. Our main job is to process complex image data, whether big or small, into digestible information in the form of quality figures, tables, or graphs (Giepmans et al. 2023). Presently, we collect data at an unseen pace and in such vast file size resulting in challenges to curate, store, handle, and hence realistically analyse. This has led researchers to make their data publicly available (read, open-source) and invite colleagues to use it for further research (read, data mining), thereby promoting collaboration and transparency and allowing scientists to contribute to, leverage, and build on existing knowledge (Hartley et al. 2022). As a result, data cleansing (also known as data hygiene) solutions are increasingly important, including the development of compatible open-source software tools and international standards for managing, sharing, and analysing data across various platforms and research environments (Poger et al. 2023).

There is not a single microscopy meeting, workshop, summit, or brainstorming session—whether face-to-face, via video conferencing, or in-person—technical terms related to big data are used appropriately or incorrectly in the conversation, such as “Graphical Processing Units (GPUs)”, “Edge Computing”, “Cloud & Hybrid Solutions”, “Data movement”, “Deep & Machine Learning”, “Explainable Artificial Intelligence (EAI)”, “ChatBots”, “Text-to-Image”, “Trusted Research Environment (TRE)”, “Large Language Models (LLMs)”, and “High Performance Computing (HPC)”. During those gatherings, everyone lately seems to be a microscopy data expert, confidently pontificating with a ready-to-go, all-encompassing solution for any data problem, big (data) or small (data). This is precisely the challenge we face. Often we—i.e. “wet lab microscopists”—are called upon to advise on data analysis and data management matters but may fall short due to limited educational background in these areas. Although we wet lab microscopists take pride in our proficiency with designing imaging experiments, collecting data, and using custom-made image analysis software tools, we must resist the temptation to believe that we possess all-encompassing wisdom, as our expertise primarily lies in experimentation and imaging rather than in the fine details of data management and analysis.

Furthermore, it is important to note that the data challenge mentioned above does not solely arise from wet lab microscopists; it also stems from the misleading impression created by manufacturers, who often suggest that their microscope solutions can automatically manage all aspects of data processing without requiring expertise in image data analysis from the microscopist. This includes user-friendly software features like sliders that provide little guidance and lack clear indications of the artefacts they could produce and/or potentially hampering further complex post-data processing and analysis. As such, the resulting danger of scientific findings derived from artefacts from vendor-ready solutions is very real, especially when proprietary image formats and/or black-box artificial intelligence applications are used, which unfortunately can lead to rushed or incorrect conclusions. Selected examples of recommended reading on the challenges in deep learning, the pitfalls of commercial microscopy analysis workflows compared with open-source software solutions, and the limitations of proprietary image formats can be found in the following references (Hoffman et al. 2021; Haase et al. 2022; Cimini 2024).

From the aforementioned paragraphs, it is clear that we need a specialist in this domain. Consequently, a “dry lab microscopist” would be an ideal candidate for the role. This position would bridge the gap between the expertise of the wet lab microscopist and the sophisticated data management needs, ensuring both more precise and insightful research outcomes (Braet and Ratinac 2007). A similar call was made in a contribution to Nature by Assaf Zaritsky, where the term “dry cell biologist” was coined (Zaritsky 2016). We could also refer to them as “prompt microscopist”, as the job description for this type of microscopist would require the specific high-level acquired knowledge to handle command prompts, system prompts, artificial intelligence prompts, and prompts related to data interoperability and lifetime data retention. And actually, that is exactly what we need moving forward: i.e. dedicated scientists or support staff devoted to the art of handling and managing all aspects of microscopy data (see next paragraph). Also, there is a point made by Jones and Strange in which they use “research software engineers” to describe scientists supporting and developing solutions to digital microscopy (Jones and Strange 2023). The content that follows aims to define what we mean by “dry lab microscopist” and, more specifically, what the typical job requirements are for these individuals.

**Fig. 1:**
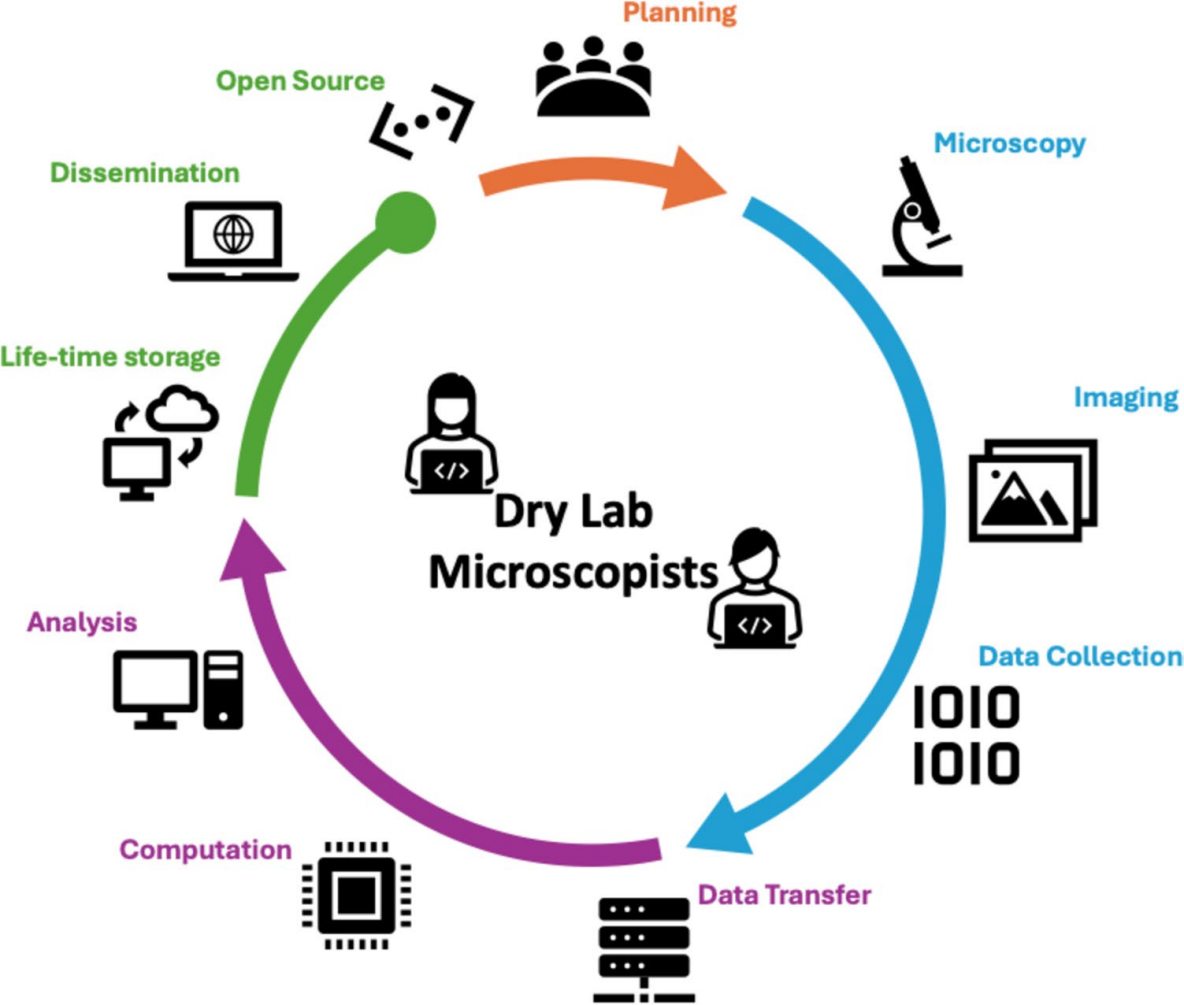
Diagram depicting the various contributions of the dry lab microscopist, who plays an enabling role in modern microscopy-driven research. The specialist contributes to the research project by providing support throughout the entire data cycle. As such, this support covers all stages of the project, including planning (orange), data acquisition (blue), and data processing (purple). Furthermore, the specialist also contributes to data archiving, assists in the preparation of data sets for publication to ensure they meet the highest scientific standards (green), and provides support to ongoing access to research data (black dotted double arrow). Note that the schematic drawing above is based on the ‘research user experience’ training and support model at Sydney Microscopy & Microanalysis, University of Sydney. This research user cycle has been implemented since the early 2000s as a good practice for training microscopists (Ratinac and Eichhorn 2006; Braet and Ratinac 2007)

Key tasks that should typically be covered by the dry lab microscopist include developing and applying various software and computational tools to microscopy data, such as image reconstruction, biophysical simulations, image segmentation, data visualisation, and quantitative analysis. They should also provide guidance and solutions for handling datasets and long-term storage, developing methods for real-time data acquisition and analysis (on the fly), and leveraging AI for image analysis and pattern recognition. Additionally, they should discuss any data and microscopy limitations in the planning phase that might impede the study’s objectives and affect data quality. Figure 1 schematically depicts the various roles and their contributions as enablers to modern microscopy-oriented research. There should be no doubt that dedicated professional data scientists are not a luxury but rather a necessity, essential for driving quality-driven microscopy science and ensuring that the analysis, reporting, and dissemination of data conform to the best practices (Bals et al. 2024).

## Where are dry lab microscopists needed?

Whatever the name, browsing job opportunities on online platforms like AAS, Nature, and microscopy list servers reveals a range of related job titles all seeking individuals with expertise in bioimage informatics, computational microscopy, image data analysts, image scientists, and so on. This highlights the demand for such professionals, but the question remains: Where can we find them, and what training programs do they need to attend? Is experience more important, or is formal training crucial? Acknowledging the need is the first step, but implementing dedicated training programs—whether as part of undergraduate education or through specialised postgraduate programs—is a significant next step forward (see vide infra). Up until the last 10 or 15 years, (dry lab) microscopists fell into this role out of necessity, learning on the job for specific research projects and/or data challenges.

Nowadays, dry lab microscopists should ideally be directly embedded—or at least associated—with microscopy facilities within research institutes and universities, to address the digital data challenges that arise. These institutions must recognise and respond to the multifaceted nature of data management, analysis, and interpretation in order to effectively tackle the ever-evolving complexities of modern microscopy and standardised data handling (Schmidt et al. 2024). These specialists would ideally operate at the intersection of bioinformatics, computational biology, and structural biology, leveraging automation and artificial intelligence to drive forward our understanding of complex biological systems. While the era of collecting megabytes of image data is behind us, and terabytes and even petabytes of microscopy information are now the new ‘normal’, our capacity to appropriately handle the datasets is more pressing than ever calling for the need of a dry lab microscopist. Finally, having a dedicated expert in place will also ensure that FAIR and CARE policies are followed as standard practice and that the principles of research image data integrity are upheld.

## Advancing biophysical science through digital microscopy data

New developments in digital imaging technology in microscopy have significantly transformed biophysical research by enabling the investigation of vast amounts of data captured and processed using highly sensitive digital cameras and high-performance imaging software, alongside ongoing innovations in microscopy, computer hardware, and advanced data analysis software capabilities. As such, present-day digital imaging technology in microscopy enables enhanced image quality and the capture of qualitative and quantitative digital microscopy data, thereby facilitating the generation of meaningful scientific insights and conclusions. Selected examples of microscopy and digital imaging in biophysics include 3D structural analysis of biomolecules, such as proteins and organelles, using techniques like high-throughput cryo-electron microscopy with direct electron detection (Ziemianowicz and Kosinski 2022). It has also facilitated high-content observation of dynamic cellular processes in 4D (X, Y, Z, and t), using advanced laser microscopy to track molecular interactions within living cells, tissues, and even at the organismal level (Balasubramanian et al. 2023). Another relevant example involves the application of atomic force microscopy approaches combined with simulation tools that allow real-time biophysical insights into the macromolecular properties and three-dimensional conformational dynamics of proteins and their interactions with various matter at the atomistic level (Amyot et al. 2023).

Each of the selected examples given above has a strong focus on understanding the (bio)physical principles underlying image formation, probe behaviour, and sample understanding to ensure high-quality data are acquired, and, hence, accurate models with statistical rigor are obtained. Indeed, structural biology benefits greatly from the dry lab microscopist’s expertise, as high-resolution imaging techniques, such as cryo-electron microscopy, produce vast amounts of structural data. Here, the dry lab microscopist’s role is crucial in translating these data into meaningful biological insights through computational analysis and modelling. This process not only helps in understanding molecular structures but also in predicting their functions and interactions with potential new drug compounds. Likewise, the contribution of dry lab microscopists to automation and artificial intelligence solutions has transformed the landscape in optical-based imaging. By applying algorithms and deep learning tools, they can decipher complex biological information within vast amounts of data that were previously too cumbersome and time-consuming for traditional analysis methods. This advancement allows for the identification of patterns and correlations in microscopy data that were once elusive. For scanning probe microscopy, collecting calibrated data on the fly at unprecedented speeds accelerates discovery, opening up an entirely new and exciting field of biophysical pathology (i.e. biophysical models of disease).

## What are the best university programs for aspiring dry lab microscopists?

There is no single answer to this question. To the best of our knowledge, there is not a single specific program designed to train undergraduate or postgraduate students to become well-rounded data scientists in microscopy, as described herein. The same is true for the training of wet lab microscopists (Wright et al. 2024). From experience, much of the data work is currently managed by wet lab microscopists who have a keen interest in digital data challenges and pursue upskilling through self-teaching (Braet and Taatjes 2024). There is essentially nothing wrong with this approach, except that we often fall short in typical computing, programming, and information technology support. The second group of colleagues, who enter the job with relevant undergraduate backgrounds in computing, mathematics, software engineering, or digital science, often lacks the necessary microscopy or biosciences background to fully understand and address the challenges related to microscopy, imaging, and data.

Given this knowledge and in the absence of a dedicated degree program, undergraduates who wish to pursue a career as dry lab microscopists should aim for a double or combined degree. This could involve an undergraduate life sciences degree combined with postgraduate education in data science, or conversely, an undergraduate degree in computer science followed by postgraduate studies in life sciences that include practical microscopy research components. Indeed, current higher education programs are beginning to address market demand by offering degrees in data science, such as Graduate Certificates or Diplomas in Data Science, Master’s degrees in Applied Data Science or Data Analytics, and research-focused Master’s or PhDs in Computer Science. The training duration for these programs varies, ranging from 6 to 36 months. By following any of the above options, students will gain a strong foundation in big data analytics and machine learning/artificial intelligence and will be trained on state-of-the-art contemporary tools to analyse large amounts of diverse data to uncover hidden patterns, unknown correlations, and other valuable insights. By selecting one of these data science degree programs alongside educational options in microscopy sciences, an interested student can be well-prepared to pursue a career as a dry lab microscopist by following one of the training options above.

In the ideal world, the training of dry lab microscopists must include wet lab practice. The insights gained from this combined educational approach is crucial for establishing an understanding of what wet lab scientists are attempting to achieve and enables dry lab scientists to effectively compliment subsequent data collection and analysis required to draw accurate and relevant conclusions. Indeed, without this interplay between wet and dry lab microscopists, the temptation to believe in an all-encompassing wisdom may prevail, whether it originates from the wet lab or the dry lab microscopists.

## Conclusion, consideration, and outlook

The dry lab microscopist plays, and will play, a crucial role in any well-sized microscopy facility and should be the key image data expert and first port of call, bringing extensive experience in designing, managing, and supporting the digital microscopy environment. This includes providing support for hardware and offering advice on the most appropriate and applicable analysis software solutions. As such, this expert will collaborate closely with researchers to design and implement the appropriate data workflows for their specific imaging needs, ensuring that high standards are maintained.

A somewhat intriguing observation is that successful and highly regarded journal titles that began to emerge in the 1990s and early 2000s, such as “Biological Imaging” and “Bioimaging”, and which were devoted to reporting on developments and applications in digital imaging and microscopy, have ceased to exist. This has created, to a certain degree, a void for microscopists trying to stay up-to-date with the latest advancements in the field. There is no clear explanation for this, and one might have expected the opposite considering how digitalisation thrives microscopy science. Fortunately, interdisciplinary journals—without naming them—have now implemented dedicated sections covering contributions on these emerging topics. Alternatively, troubleshooting-focused and (interactive) web-based platforms have gained significant acceptance among researchers, including microscopists, seeking collaborative solutions to complex data problems. Open access platforms (e.g. GitHub, Fiji, FocalPlanes, and ResearchGate) serve as great resources which enable users to pose questions, share insights, scripting code, and troubleshoot issues quickly.

As technology continues to evolve, the role of the dry lab microscopist will undoubtedly expand, providing new insights and possibilities in our quest to understand the living world. For the near future, much can be expected about the integration of AI into data analysis that requires continuous development of algorithms by the dry lab microscopist to ensure accuracy and relevance. Machine learning models, on the other hand, will increasingly have a bigger footprint in automated image analysis, reducing the time required to process thousands of samples (and, digital data) and enabling researchers to focus on interpreting results and generating sound conclusions instead. The opportunities are immense. Watch the space (and specialists) to come!

## Data Availability

No datasets were generated or analysed during the current study.
